# Plants utilise ancient conserved peptide upstream open reading frames in stress‐responsive translational regulation

**DOI:** 10.1111/pce.14277

**Published:** 2022-02-15

**Authors:** Barry Causier, Tayah Hopes, Mary McKay, Zachary Paling, Brendan Davies

**Affiliations:** ^1^ Faculty of Biological Sciences, Centre for Plant Sciences University of Leeds Leeds UK; ^2^ Faculty of Biological Sciences, School of Molecular and Cellular Biology University of Leeds Leeds UK

**Keywords:** 5′‐leader, 5′‐UTR, abiotic stress, Arabidopsis, CPuORF, ribosome stalling, translation regulation, uORF

## Abstract

The regulation of protein synthesis plays an important role in the growth and development of all organisms. Upstream open reading frames (uORFs) are commonly found in eukaryotic messenger RNA transcripts and typically attenuate the translation of associated downstream main ORFs (mORFs). Conserved peptide uORFs (CPuORFs) are a rare subset of uORFs, some of which have been shown to conditionally regulate translation by ribosome stalling. Here, we show that Arabidopsis CPuORF19, CPuORF46 and CPuORF47, which are ancient in origin, regulate translation of any downstream ORF, in response to the agriculturally significant environmental signals, heat stress and water limitation. Consequently, these CPuORFs represent a versatile toolkit for inducible gene expression with broad applications. Finally, we note that different classes of CPuORFs may operate during distinct phases of translation, which has implications for the bioengineering of these regulatory factors.

## Summary statement

We describe three ancient CPuORFs that conditionally regulate translation of any downstream ORF and show the utility of these CPuORFs for rapid responses to agronomically significant stress conditions.

## 1 INTRODUCTION

**Figure 1:**
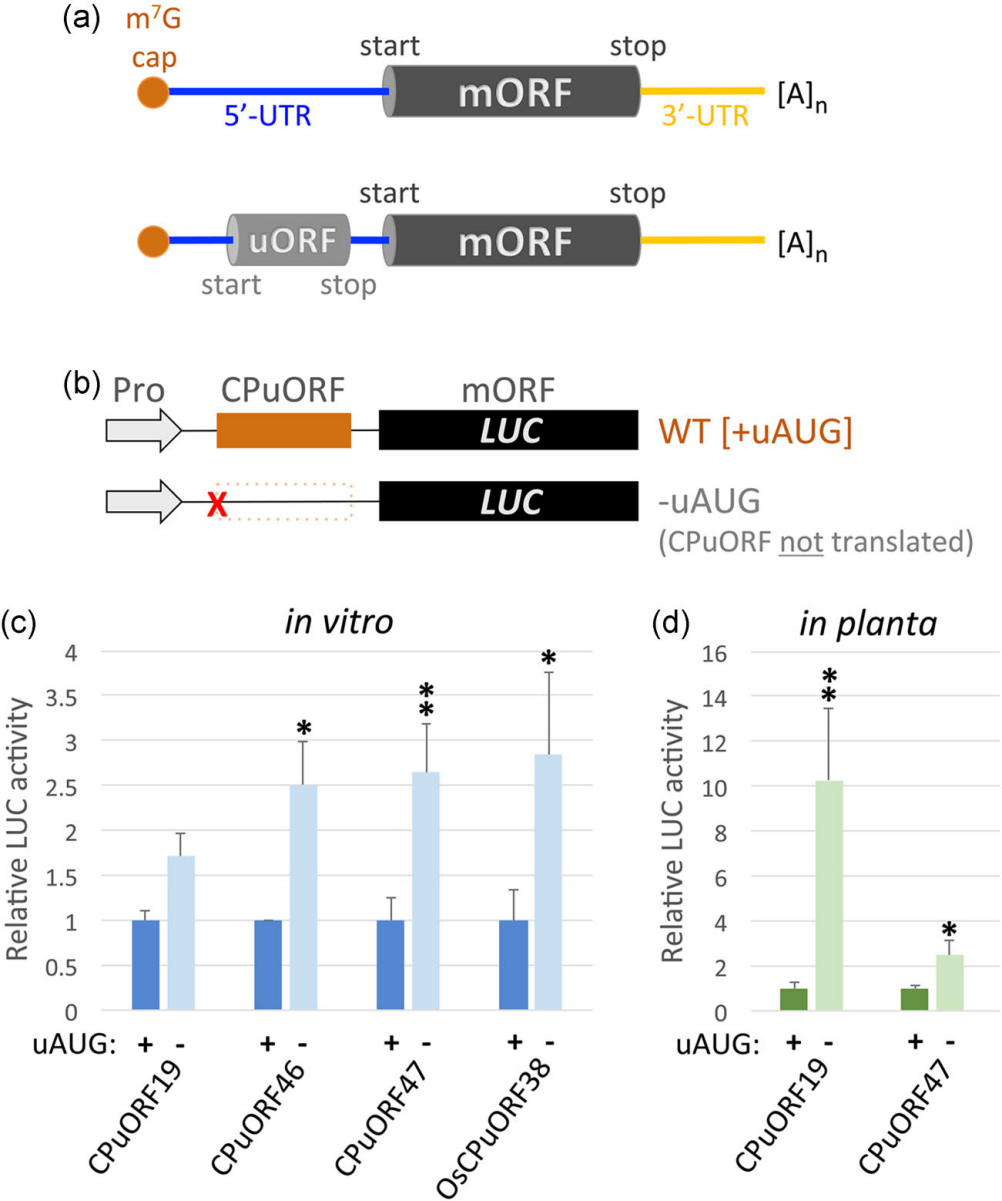
Plant CPuORFs attenuate activity of downstream ORFs. (a) Illustration of typical eukaryote transcripts. Top: textbook transcript with 5′‐m^7^G cap (red), a 5′‐untranslated region (5′‐UTR; blue), the major open reading frame (mORF; dark grey) of the transcript, which encodes the main protein product, a 3′‐UTR (yellow) and the poly‐A tail ([A]_n_) at the 3′‐end of the transcript. Bottom: a transcript with a protein‐encoding upstream open reading frame (uORF; light grey) within the 5′‐UTR. (b) Summary of constructs used. Arrows represent the promoter (SP6 for in vitro assays, 35S in planta). Lines represent the 5′‐UTR. The black box represents the major open reading frame (mORF), which encodes the luciferase (LUC) reporter. The coloured box represents the various CPuORFs studied. The dotted box represents CPuORFs where the start codon (uAUG) has been mutated (red cross) to prevent CPuORF translation, releasing the inhibition of mORF translation. (c) LUC activity measured in wheat germ extracts charged with mutant CPuORF (−uAUG) reporters, relative to the appropriate WT control (CPuORF + uAUG). Data represent the mean ± SEM of three biological replicates of the experiment. (d) LUC activity measured in leaves of transgenic Arabidopsis plants transformed with mutant CPuORF (−uAUG) reporters, relative to the appropriate WT control (CPuORF + uAUG). Data represent the mean ± SEM (for CPuORF19 + uAUG the number of independent lines (*n*) = 17, CPuORF19 − uAUG *n* = 16, CPuORF47  + uAUG *n* = 22, CPuORF47 − uAUG *n* = 18). CPuORF, conserved peptide upstream open reading frame; HSD, honestly significant difference; UTR, untranslated region; WT, wild‐type. In (c) and (d) significant differences between the +uAUG and −uAUG reporter for each CPuORF at *p < 0.05 and **p < 0.01 (Tukey HSD inference) [Color figure can be viewed at wileyonlinelibrary.com]

Posttranscriptional regulation is a critical means of controlling protein levels. It provides a mechanism to achieve rapid responses to both internal and external stimuli, without the requirement to initiate or repress transcription (Ingolia, 2016; H. Zhang et al., 2019). In sessile organisms such as plants, the ability to respond immediately to an ever‐changing environment is key to normal growth and development. The untranslated regions (UTRs) of transcripts have been implicated in the control of translation. In particular, up to 50% of transcripts from animals, fungi and plants contain potentially translatable upstream open reading frames (uORFs) within their 5′‐UTRs (also known as 5′‐leader sequence) (T. Zhang et al., 2020) (Figure 1a). Typically, uORFs attenuate translation of the downstream major open reading frame (mORF), which encodes the main protein product of the transcript (Barbosa et al., 2013; Johnstone et al., 2016; Kurihara, 2020; von Arnim et al., 2014). uORF‐mediated translation inhibition can either occur passively, as ribosomes dissociate after uORF translation or by an active mechanism, in which uORF translation causes ribosome stalling (Kurihara, 2020; von Arnim et al., 2014). Ribosomal arrest may sequester translating ribosomes, blocking their access to downstream ORFs, or may be interpreted as abnormal translation termination, triggering transcript destruction through pathways such as nonsense‐mediated messenger RNA decay (NMD) (Lloyd, 2018; Yamashita, 2013). In either case, translation of the downstream mORF is inhibited.

Numerous studies have demonstrated that the ribosome exit tunnel plays an important role in regulating translation (Bhushan et al., 2010; Liutkute et al., 2020). Ribosome stalling during uORF translation is a common regulatory mechanism that operates in prokaryotes and eukaryotes, to control translation of downstream ORFs. Structural analyses of ribosomes translating uORF regulatory peptides reveal that direct interactions between the nascent peptide and the ribosome exit tunnel cause ribosome stalling (Bhushan et al., 2010: Seidelt et al., 2009). uORF‐mediated translational regulation has been shown to be conditional in some cases, where specific small molecules determine whether translation is turned on or off (Seip & Innis, 2016). It is emerging that translating uORF peptides, which cause ribosome stalling, can act as receptors for small effector molecules, either within the ribosome exit tunnel or via an extra‐ribosomal domain (Ito & Chiba, 2013; von Arnim et al., 2014). Such effectors are diverse and include antibiotics and metabolites in bacteria, and polyamines, amino acids and *S*‐adenosylmethionine in eukaryotes (Ito & Chiba, 2013).

uORF‐mediated ribosome stalling is dependent upon the sequence of the nascent peptide (Seip & Innis, 2016). Despite this, most uORFs prevalent in eukaryotic transcripts do not appear to be under selective pressure to conserve their encoded amino acid sequence. However, there is a rare subset of uORFs where peptide sequence is conserved over large evolutionary distances, suggesting functional significance. In plants, genome‐wide comparisons between Arabidopsis (*Arabidopsis thaliana*), rice (*Oryza sativa*) and other angiosperms has led to the identification of predicted transcripts containing one or more conserved peptide uORFs (CPuORFs), including at least 123 transcripts in Arabidopsis (Hayashi et al., 2017; Hayden & Jorgensen, 2007; Jorgensen & Dorantes‐Acosta, 2012; Takahashi et al., 2012, 2020; Tran et al., 2008; van der Horst et al., 2020; van der Horst et al., 2019; Vaughn et al., 2012) (for a database of Arabidopsis CPuORFs, with details on the function and evolution of these elements, see Table S1). Comparison of CPuORF peptide sequences from diverse angiosperm species places them into over 150 homology groups (HGs) (Takahashi et al., 2020). Interestingly, a proportion of CPuORFs discovered in angiosperms have also been identified in earlier diverging plants, including bryophytes and green algae, suggesting that these short sequences have been maintained for an extraordinary length of time (Hayden & Jorgensen, 2007; Takahashi et al., 2020) (Table S1). In addition, based on patterns of sequence conservation, CPuORFs can be divided into two broad classes: class I is characterised by a highly conserved C‐terminal region, while in class II the entire CPuORF peptide sequence or the N‐terminal and/or middle regions are conserved (Takahashi et al., 2012) (Table S1). Several plant CPuORFs have been shown to inhibit translation of downstream ORFs (Alatorre‐Cobos et al., 2012; Ebina et al., 2015; Hanfrey et al., 2005; Laing et al., 2015; Rahmani et al., 2009; Ribone et al., 2017; Takahashi et al., 2020; Zhu et al., 2012), which in some cases is caused by ribosome stalling on the CPuORF (Bazin et al., 2017; Hayashi et al., 2017; Ribone et al., 2017; Uchiyama‐Kadokura et al., 2014; Yamashita et al., 2017). Further, we previously identified CPuORFs as a trigger of NMD in plants (Lloyd & Davies, 2013; Rayson et al., 2012).

The biological functions of most plant CPuORFs are not known, although they are generally associated with mORFs encoding regulatory proteins (Hayden & Jorgensen, 2007; Jorgensen & Dorantes‐Acosta, 2012). For the handful of plant CPuORFs that have been functionally characterised, they act as conditional regulators of translation, responding to a range of different signals, including sucrose, ascorbate, phosphocholine, polyamines, galactinol, heat stress, pathogen attack and photosynthetic signals (Alatorre‐Cobos et al., 2012; Bazin et al., 2017; Guerrero‐González et al., 2016; Hanfrey et al., 2005; Imai et al., 2006; Laing et al., 2015; Rahmani et al., 2009; Ribone et al., 2017; Tabuchi et al., 2006; Uchiyama‐Kadokura et al., 2014; Wiese et al., 2004; Xu et al., 2017; Zhu et al., 2012, 2018) (summarised in Table S1).

Plant CPuORFs are emerging as important posttranscriptional regulators, acting to control translation in response to changing intracellular or extracellular conditions. Indeed, the potential of CPuORFs as tools for crop improvement is now being realised (van der Horst et al., 2020; Xing et al., 2020; Xu et al., 2017; H. Zhang et al., 2018). Signal‐dependent induction of translation has potential utility in agriculture, synthetic biology and research, so here we ask whether plant CPuORFs can be used to regulate translation in response to stress conditions that are known to significantly impact global crop production. We discovered three Arabidopsis CPuORFs, annotated as CPuORF19, CPuORF46 and CPuORF47, that respond to heat stress or water limitation to regulate translation of downstream ORFs. These CPuORFs have been conserved in plants for over 350 million years of land plant evolution. Together our data demonstrate the general applicability of CPuORFs as versatile tools for inducible gene expression with applications both in the laboratory and in the field.

## 2 MATERIAL AND METHODS

### 2.1 Plant growth

Standard plant growth for Arabidopsis wild‐type (Col‐0), *upf2‐10* NMD‐mutant (Merchante et al., 2015) or transgenic lines (Col‐0 background) was on compost at 20°C, under long days (16 h light/8 h dark).

### 2.2 Plasmid construction

All oligonucleotides used in this study are described in Table S2. To clone Arabidopsis or rice CPuORF‐containing 5′‐UTRs, total RNA was prepared from leaves using the Qiagen Plant RNeasy Mini Kit, according to the manufacturer's instructions (Qiagen). cDNA was prepared from total RNA (1 μg) using the SuperScript II reverse transcriptase (Life Technologies). All plant constructs were transformed into Agrobacterium (*Agrobacterium tumefaciens*) strain GV3101, and Arabidopsis plants were subsequently transformed using the floral dip method (Clough & Bent, 1998). Transgenic seed was selected using the green fluorescent protein seed‐coat marker provided on the pALLIGATOR3 vector (Gateway modified pFP101; Bensmihen et al., 2004).

For the in planta constructs, 5′‐UTRs were PCR (polymerase chain reaction) amplified using Phusion DNA polymerase (Thermo Fisher Scientific) and appropriate primers containing attB1 (forward) or attB5r (reverse) Gateway cloning sites. Subsequently, 5′‐UTR sequences were Gateway cloned into the pDONR P1‐P5r vector (Invitrogen). The Luciferase (LUC) reporter gene was PCR amplified from the ‘Luciferase SP6 Control DNA’ vector (Promega) using forward (attB5‐LUCIFERASE) and reverse (nLUC‐B2‐R) primers containing attB5 and attB2 Gateway sites, respectively. The LUC fragment was subsequently Gateway cloned into the pDONR221 P5‐P2 vector (Invitrogen). 5′‐UTR and LUC sequences were assembled using MultiSite Gateway technology into plasmid pALLIGATOR3 to generate 35S:CPuORF–LUC constructs. To generate constructs in which the translation initiation codon of the CPuORF was mutated, new forward primers were designed containing the desired mutation (with attB1 site; Table S2). The new forward primer was used in combination with the appropriate reverse primer for PCR amplification using the relevant wild‐type pDONR221 P1‐P5r construct as template. The mutated PCR product was cloned into pDONR221 P1‐P5r and assembled with the LUC gene (in pDONR221 P5‐P2) into pALLIGATOR3.

In vitro LUC reporters were made in one of two ways. Firstly, the CPuORF alone was amplified from the relevant pALLIGATOR3 construct, using a CPuORF‐specific forward primer that also contained a Kozak sequence (to ensure efficient translation of the CPuORF in the heterologous in vitro system; AACAGACCACCAUG, translation initiation sequence underlined) with a *Hind*III restriction site for cloning, together with a CPuORF‐specific reverse primer containing a *Not*I site for cloning (Table S2). PCR products were *Hind*III‐*Not*I digested and ligated into the ‘Luciferase SP6 Control DNA’ vector, digested with the same restriction enzymes, to generate SP6:CPuORF‐LUC constructs where the CPuORF was in a nonnative 5′‐UTR context. As above, versions of these constructs were made in which the start codon of the CPuORF was mutated, through the use of modified forward primers (Table S2). Purified plasmids were used as templates in transcription/translation reactions as described below. Secondly, to test the CPuORF in its native 5′‐UTR context in vitro, the 5′‐UTR containing the CPuORF and the LUC gene was amplified from the relevant plant pALLIGATOR3 construct, using a forward CPuORF‐specific primer with a tail containing the Kozak sequence and the SP6 promoter sequence, together with a reverse primer (LUC‐TNT‐R) specific to the 3′‐end of the LUC gene (Table S2). Purified PCR products were used directly in transcription/translation reactions as described below.

Mutagenesis of the C‐terminal domain of CPuORF47 was carried out using the Q5 site‐directed mutagenesis kit, following the manufacturer's instructions (NEB). PCR reactions were performed using the 47SDM primer pairs listed in Table S2, with SP6:CPuORF47‐LUC (in vitro) or pDONR221[P1‐P5r]‐CPuORF47 (in planta) as templates. Following sequence validation, SP6:CPuORF47^sdm1‐sdm3^‐LUC constructs were used directly in in vitro LUC assays, while pDONR221[P1‐P5r]‐CPuORF47^sdm1‐sdm3^ constructs were assembled together with the LUC reporter gene into pALLIGATOR3 using Multisite Gateway for plant assays.

### 2.3 Luciferase reporter assays

For in vitro assays, purified PCR products (250 ng) or plasmid constructs (500 ng) were transcribed and translated using the TNT SP6 High‐Yield Wheat Germ Master Mix. Reactions (25 μl) were assembled and incubated for 2 h at 25°C, according to the manufacturer's instructions (Promega). TNT reactions were performed in triplicate. To measure luciferase activity, 10 μl of each TNT reaction was mixed with an equal volume of LUC assay buffer (0.5 mM luciferin, 0.01% (w/v) Triton X‐100) in an opaque 96‐well plate, and bioluminescence detected using the LB985 NightShade Plant Imaging System (Berthold), with the following settings: exposure 0.1–1 s, 4×4 binning, gain high, slow read out. Photon counts per second (cps) were measured for each sample and averaged between replicates.

For in planta assays, fully expanded, healthy leaves were selected from the rosettes of transgenic plants, incubated in LUC assay buffer for 5 min at room temperature (RT) and placed in the dark for 5 min. Bioluminescence was measured as above, except that exposure was 60 s. Cps/mm^2^ of leaf was calculated and averaged between replicates. To normalise for possible differences in transgene expression (due to positional effects or CPuORF‐mediated transcript decay) we analysed multiple independent T_1_ plants for each construct (for details see Table S3).

### 2.4 Stress and chemical treatments for in planta LUC assays

To test for response to water limitation in a controlled manner, leaves from transgenic plants were incubated in 0.5xMS liquid medium containing 300 mM mannitol for 24 h at RT. As control, leaves from the same plants were incubated in 0.5xMS for 24 h at RT.

To test for response to increased temperatures, leaves from transgenic plants were incubated at 37°C for 6 h on damp filter paper. As control, leaves for the same plants were incubated at RT for 6 h on damp filter paper.

To test for responses to thermospermine, leaves from transgenic plants were incubated in 0.5xMS liquid medium containing 0.5 mM thermospermine for 24 h at RT. As control, leaves from the same plants were incubated in 0.5xMS for 24 h at RT.

### 2.5 Bioinformatics

PARE (parallel analysis of RNA ends) data was taken from the GEO database under the accession number GSM280226 (German et al., 2008). PARE reads for each CPuORF transcript were retrieved and analysed using the Arabidopsis Next‐Gen Sequence DBs (mpss.danforthcenter.org/dbs/index.php?SITE=at_pare). Global analysis of PARE reads associated with *Arabidopsis* CPuORFs was originally published by Hou et al. (2016), which we manually inspected for class I and class II CPuORFs.

## 3 RESULTS

### 3.1 CPuORF19 and HG17 CPuORFs act as autonomous regulators of mORF translation

Here, we investigated Arabidopsis CPuORFs belonging to two previously uncharacterised HGs. The first is HG7a and its sole representative CPuORF19 (a class II CPuORF from *At1g36730*), which has been conserved at least since bryophytes and angiosperms diverged almost 0.5 billion years ago (Lloyd & Davies, 2013; see Figure S1a). The second is HG17, which has four members in Arabidopsis, CPuORF45, 46, 47 and 48, although here, we focus on CPuORF46 (*At3g53400*) and CPuORF47 (*At5g03190*). For an evolutionary perspective, we also selected a representative member of HG17 from rice (OsCPuORF38 from *LOC_Os02g52300*). HG17 CPuORFs can be found in fern sequences (Takahashi et al., 2020; see Figure S1b), suggesting that these have been conserved for at least 350 million years of plant evolution.

As the paradigm is that translation of a uORF/CPuORF is required for translational repression, we compared the activity of the wild‐type CPuORFs with that of altered versions in which the translation initiation codon (uAUG) of the CPuORF was mutated to prevent its translation in a *LUCIFERASE* (*LUC*) reporter gene assay (Figure 1b). However, we previously demonstrated that CPuORF‐containing transcripts are common substrates of NMD (Rayson et al., 2012), and we show here that NMD frequently suppresses the abundance of CPuORF‐containing transcripts (Figure S2 and Table S3). We suggest that NMD is always considered when designing experiments to examine the activity of CPuORFs. Consequently, we first measured reporter activity in a cell‐free expression system to establish whether these CPuORFs act as inhibitors of translation in the absence of NMD. As shown in Figure 1c (see also Table S3), all reporters in which the uAUG was mutated had increased LUC activity relative to WT versions, indicating that translation of the CPuORF is necessary to attenuate translation of the associated mORF. We also tested CPuORF19 and CPuORF46 (±uAUG) in their native 5′‐UTR context. As above, reporters with a mutated uAUG had significantly higher LUC activity relative to WT constructs, and to a similar level observed with the nonnative 5′‐UTR (Table S3), demonstrating that the information required for CPuORF‐induced attenuation of mORF translation is confined to the CPuORF.

To verify that both classes of CPuORFs also function as translational regulators in planta, we tested CPuORF19 and CPuORF47 and found that, relative to the WT constructs; those with a mutated uAUG had significantly higher levels of LUC activity (Figure 1d and Table S3), closely mirroring the in vitro data.

Taken together, the data shows that in common with other previously studied CPuORFs, translation of HG7a and HG17 CPuORFs inhibits translation of downstream ORFs. It should be noted that the HG17 CPuORF47 transcript may undergo alternative splicing in Arabidopsis cells, resulting in an ORF that runs continuously between the CPuORF and the mORF (thus HG17 is also known as alternative N‐termini (aNT) family aNT25; Hayden & Jorgensen, 2007; Jorgensen & Dorantes‐Acosta, 2012). Several pieces of evidence indicate that alternative splicing does not influence the data produced for CPuORF47, and include (1) the putative splice acceptor site in *At5g03190* is within the predicted mORF coding region, which we have replaced with the heterologous *LUC* reporter gene in our constructs, (2) the uORF‐mORF fusion ratio of HG17 transcripts is below the 0.3 thresholds for ‘spurious’ CPuORFs (Takahashi et al., 2020), suggesting that HG17 genes encode true CPuORFs, and (3) our in planta data matches that from the in vitro assays in which splicing does not occur.

### 3.2 HG17 CPuORFs repress translation in a peptide sequence‐dependent manner

**Figure 2:**
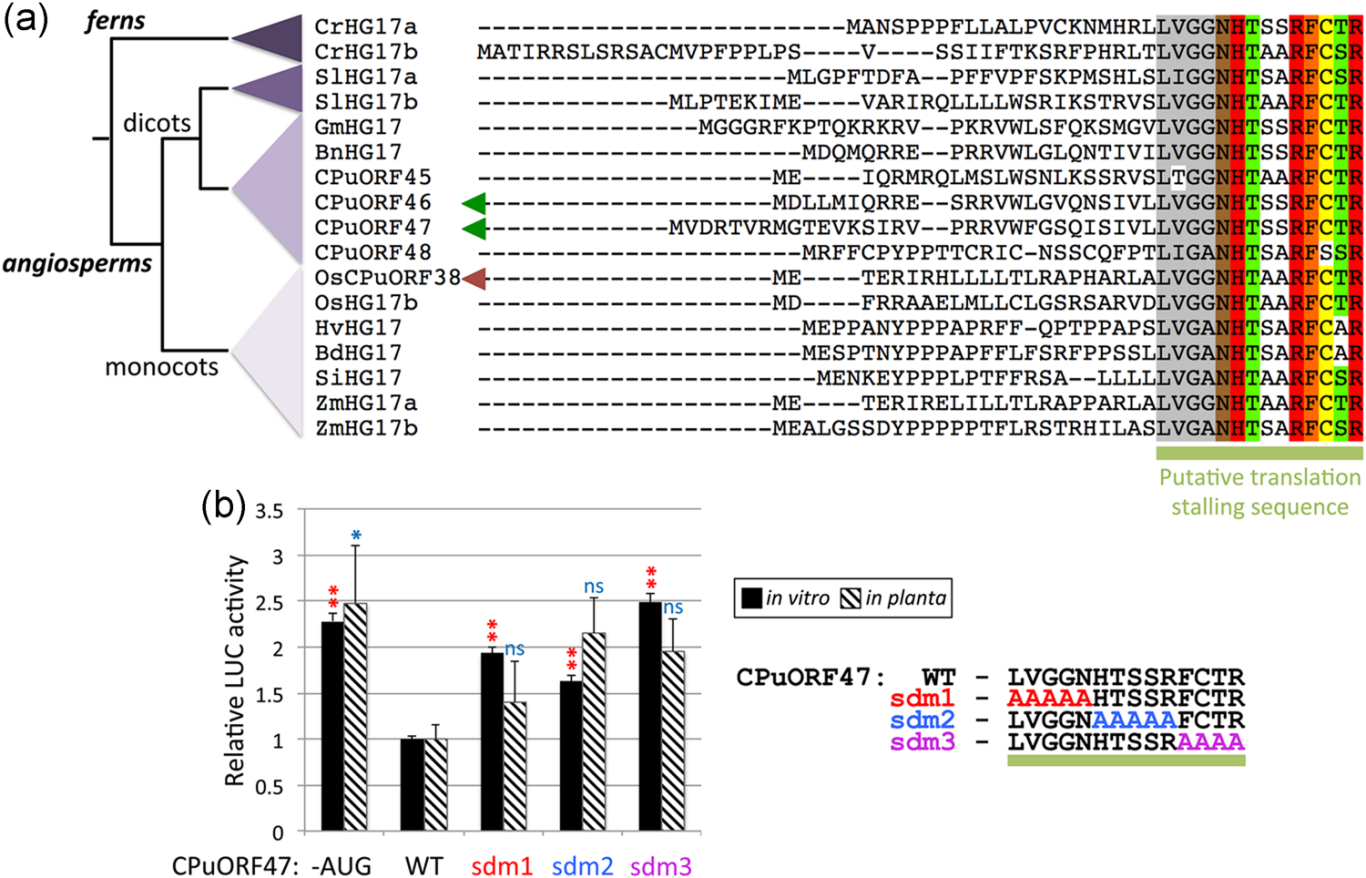
HG17 CPuORFs function in a peptide sequence‐dependent manner. (a) Clustal Omega alignment of HG17 family CPuORF peptide sequences from across the land plant phylogeny (shown in the cladogram to the left). Aligned sequences are from the following species: *Ceratopteris richardii* (Cr), *Solanum lycopersicum* (Sl), *Glycine max* (Gm), *Brassica napus* (Bn), *Arabidopsis thaliana* (CPuORF45‐48), *Oryza sativa* (OsCPuORF38 and OsHG17b), *Hordeum vulgare* (Hv), *Brachypodium distachyon* (Bd), *Setaria italica* (Si) and *Zea mays* (Zm). Arrows indicate the Arabidopsis (green) and rice (red) HG17 CPuORFs investigated in this study. The green bar below the alignment highlights the highly conserved residues that are likely to include sequences that cause translation stalling. (b) Chart comparing LUC activity for CPuORF47 CPuORF reporters mutated within the conserved C‐terminus (sdm1–3; mutations shown in alignment to the right) relative to appropriate wild‐type constructs. For in vitro (black bars), the data represent the mean ± SEM of three biological replicates. For in planta (striped bars) data represent means ± SEM (for CPuORF47 − uAUG the number of independent lines (*n*) = 18, CPuORF47 + uAUG (WT) *n* = 22, sdm1 *n* = 11, sdm2 *n* = 11, sdm3 *n* = 12). CPuORF, conserved peptide upstream open reading frame; HSD, honestly significant difference; LUC, luciferase; WT, wild‐type. Significant differences between WT and mutant CPuORFs are indicated (*p < 0.05 and **p < 0.01; Tukey HSD inference) [Color figure can be viewed at wileyonlinelibrary.com]

HG17 are class I CPuORFs, which are highly conserved at the C‐terminus (Arabidopsis HG17 CPuORFs share >57% identity across the C‐terminal 14aa of the peptide), but more divergent at the N‐terminus of the encoded peptide (the Arabidopsis HG17 peptides share no overall identity outside the C‐termini) (Figure 2a). To examine the functional relevance of the conserved C‐terminus of these peptides we generated a series of mutations within this region of CPuORF47 (Figure 2b) and measured LUC activity both in vitro and in planta relative to the WT constructs. We found that mutations anywhere within the C‐terminus of CPuORF47 significantly reduced the effectiveness of the CPuORF to attenuate LUC activity in vitro, and to a similar level as observed for the CPuORF with mutated uAUG (Figure 2b and Table S3). Although not statistically significant, the same trend was also seen in planta (Figure 2b and Table S3), supporting our prediction that the C‐terminus of the CPuORF47 peptide includes sequences that attenuate translation of a downstream mORF.

### 3.3 CPuORFs function during distinct phases of translation

**Figure 3:**
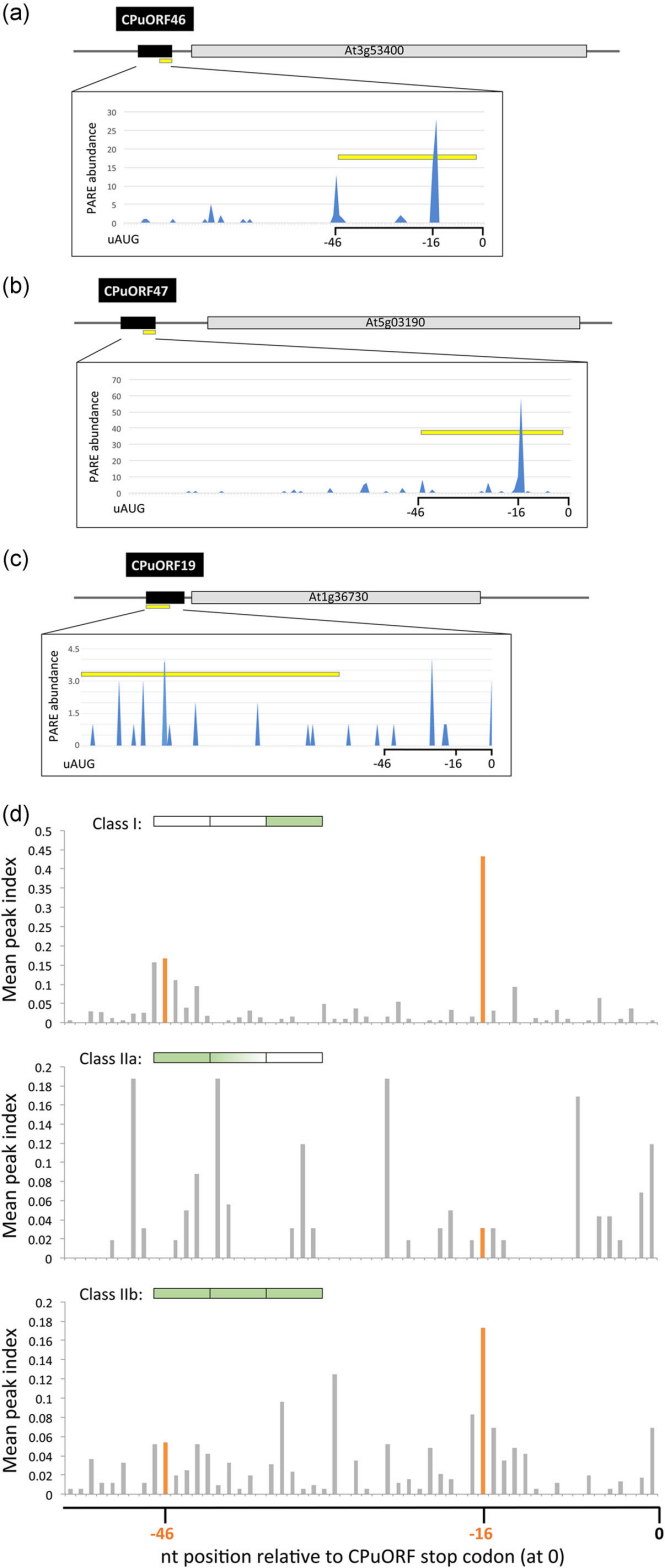
PARE read data for Arabidopsis HG17 CPuORF46 and CPuORF47 (a, b) and for CPuORF19 (c). Shown are the positional distributions of PARE reads (blue peaks) on CPuORF‐containing transcripts. Each transcript is shown diagrammatically with the CPuORF (black box) and the major ORF (grey box). PARE abundances (normalised to 10 M; German et al., 2008) are shown for the relevant CPuORF sequence. The first nucleotide of the CPuORF stop codon is assigned position 0, and nucleotide positions upstream at positions −16 and −46 are indicated. Yellow bars indicate the region on the CPuORF encoding highly conserved peptide sequences. (d) Distribution of PARE reads at the 3′‐end of class I (top), class IIa (middle) and class IIb (bottom) CPuORF coding sequences (threshold peak index >0.1). Class I CPuORFs are conserved only at the C‐terminal end of the encoded peptide, class IIa are conserved at the N‐terminal end and/or middle region of the CPuORF, and class IIb are conserved along the entire length of the peptide, as summarised diagrammatically at the top of each chart (green fill representing conservation). CPuORF, conserved peptide upstream open reading frame; PARE, parallel analysis of RNA ends [Color figure can be viewed at wileyonlinelibrary.com]

Recent ribosome profiling experiments have shown that Arabidopsis CPuORFs, including CPuORF19 and HG17 CPuORFs, are translated and cause ribosome stalling (Hsu et al., 2016; Hu et al., 2016; Juntawong et al., 2014; Liu et al., 2013). In addition, PARE data sets were shown to reflect the dynamics of ribosomes on Arabidopsis and rice CPuORFs (Hou et al., 2016). Importantly, Hou et al. (2016) discovered that many CPuORFs accumulate PARE reads at nucleotide positions −16 and/or −46 (where position 0 is the first nucleotide of the CPuORF stop codon), consistent with ribosome stalling at the termination codon. Using the same PARE data set (German et al., 2008) we observed enrichment of PARE reads at positions −16 and −46 for CPuORF46 and CPuORF47 (Figure 3a,b), although CPuORF19 showed no obvious enrichment of reads at these positions (Figure 3c).

The different patterns of PARE reads observed for the HG17 CPuORFs and CPuORF19 prompted us to look more closely at class I and class II CPuORFs to establish whether they operate during different phases of translation. The 133 Arabidopsis CPuORFs identified to date fall into 89 homology groups (Table S1), with 19 belonging to class I (representing 38 CPuORFs) and 70 to class II (95 different CPuORFs). Re‐examination of the 60 annotated CPuORFs included in the PARE analysis of Hou et al. (2016) revealed that class I CPuORFs predominantly accumulate PARE reads at nucleotide positions −16 (65.4% of class I) and −46 (42.3%) (Figure 3d and S3). By contrast, class II CPuORFs showed a more even distribution of PARE reads across the 3′‐end of the CPuORF coding sequence (Figure S3). However, class II CPuORFs can be further divided into those with a poorly conserved C‐terminus (which we term class IIa and represents ~25% of the class II CPuORFs analysed by Hou et al., 2016), and those with strong conservation at the C‐terminus of the predicted peptide (class IIb; ~75% of class II CPuORFs in the Hou et al., 2016 data set) (Figure S3). Interestingly, 34.6% of class IIb CPuORFs show a peak of PARE reads at position −16 (Figures 3d and S3). Taken together with the class I data, 50% of CPuORFs with a conserved C‐terminus accumulate PARE reads at position −16, consistent with ribosome stalling near the stop codon. For class IIa CPuORFs (of which only 12.5% have a PARE peak at −16; Figure S3), and the remainder of class I and class IIb CPuORFs with no peak at −16, the data suggests that if these CPuORFs stall ribosomes, it is not specifically at the C‐terminus.

### 3.4 Arabidopsis HG17 and HG7a CPuORFs conditionally control mORF translation in response to abiotic stress

It is known that for some plant CPuORFs, their inhibitory function can be modulated by changing intracellular or extracellular conditions (Dever et al., 2020; van der Horst et al., 2020). Arabidopsis transcripts containing HG7a or HG17 CPuORFs are up‐regulated in response to abiotic stress related to water limitation (Higashi et al., 2015; Matsui et al., 2008; Rasheed et al., 2016; Shaar‐Moshe et al., 2015; Sham et al., 2015). Here, we examined whether these CPuORFs play any additional role in a translational response to these stress conditions.

**Figure 4:**
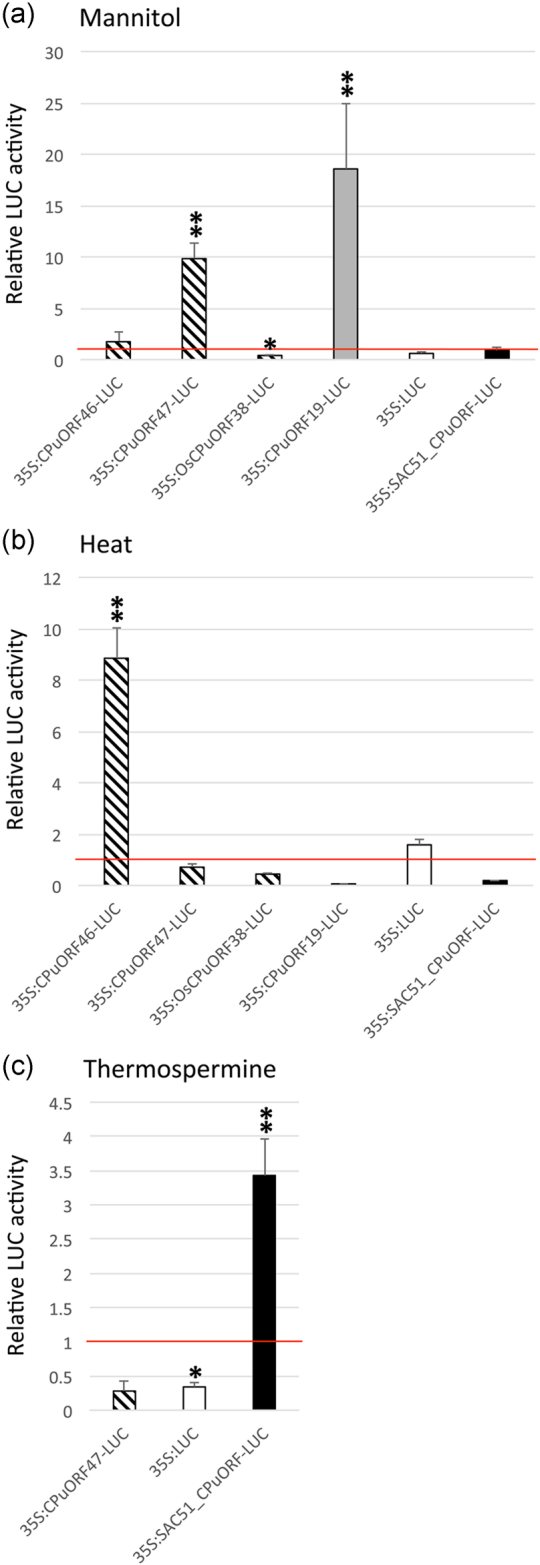
CPuORFs function as conditional regulators of translation. Translational responses following (a) mannitol, (b) heat, or (c) thermospermine treatments relative to appropriate controls are shown. Data represents means ± SEM for HG17 CPuORFs (striped bars), the HG7a CPuORF19 (solid grey bars), the HG15 SAC51_CPuORF (solid black bars), and 35S:LUC (control reporter without CPuORF; white bars). For CPuORF46‐LUC the number of independent lines (*n*) = 51, 26 and 25 for control, mannitol and heat treatments, respectively. For CPuORF47‐LUC *n* = 22 for control, mannitol and heat, and *n* = 12 for thermospermine treatment. For OsCPuORF38‐LUC *n* = 19 for all treatments. For CPuORF19‐LUC *n* = 12 for all treatments. For 35S:LUC *n* = 12 for all treatments. For SAC51_CPuORF‐LUC *n* = 21 for all treatments. CPuORF, conserved peptide upstream open reading frame; HSD, honestly significant difference; LUC, luciferase. Significant differences between controls and treatments for each CPuORF reporter at *p < 0.05 and **p < 0.01 (Tukey HSD inference) [Color figure can be viewed at wileyonlinelibrary.com]

To test whether HG17 CPuORFs mediate responses to drought‐like conditions, we compared our in planta reporter lines treated with mannitol (often used as a highly controllable drought mimic (Dubois & Inzé, 2020)) with mock‐treated samples. In response to mannitol, *35S:CPuORF47‐LUC* samples showed a dramatic increase in LUC activity relative to controls (~10‐fold; *p* < 0.01) (Figure 4a and Table S3). We also found that for *35S:CPuORF47‐LUC* plants grown in soil and subjected to drought treatment (3‐week‐old plants; water withheld for 5 days), LUC activity increased approximately 2.4‐fold relative to well‐watered controls (Table S3), suggesting that CPuORF47 is a *bona fide* posttranscriptional drought‐responsive element. To confirm that CPuORF translation was important for this conditional response, we also tested reporter lines in which the translation initiation codon of CPuORF47 had been mutated (see Figure S4a) and found that unlike the WT constructs there was no response to mannitol when CPuORF translation was inhibited (Figure S4b and Table S3). Similarly, *35S:CPuORF46‐LUC* plants also showed a response to mannitol (~1.7‐fold; Figure 4a), confirming that HG17 CPuORFs modulate translation in response to water limitation. Interestingly, however, the rice *35S:OsCPuORF38‐LUC* plants showed no mannitol response (Figure 4a), indicating that this divergent HG17 CPuORF lacks the ability to respond to drought‐like conditions, at least when expressed in Arabidopsis.

We next examined whether HG17 CPuORFs also respond to heat shock, which can induce drought‐like stress (Lamaoui et al., 2018). Although we detected no response to heat in *35S:CPuORF47‐LUC* plants, *35S:CPuORF46‐LUC* plants showed a statistically significant increase in LUC reporter activity (~8.85‐fold; *p* < 0.01) relative to controls (Figure 4b and Table S3). As with water limitation, 35S:*OsCPuORF38‐LUC* plants did not show a response to heat stress in our reporter assays (Figure 4b).

The C‐terminus of the OsCPuORF38 peptide shares significant amino acid identity with that of Arabidopsis CPuORF46 and CPuORF47 (~86%; Figure S5). In contrast, the OsCPuORF38 N‐terminus shares little overall homology with the same regions of CPuORF46 (~27%) or CPuORF47 (~18%) (Figure S5). Interestingly, the N‐terminal sequences of CPuORF46 and CPuORF47, which respond to different stress conditions, are only 50% identical (Figure S5). Together, this suggests that the different conditional responses within and between species may be mediated by the divergent N‐terminal region of these CPuORFs.

Next, we examined the conditional responses of the class II CPuORF19. We found LUC activity to be significantly elevated in *35S:CPuORF19‐LUC* plants treated with mannitol, compared to controls (~19‐fold; Figure 4a), while heat shock treatment of *35S:CPuORF19‐LUC* plants resulted in reduced LUC signal (Figure 4b). Thus, CPuORF19 is a second element identified here that regulates translation in response to water limitation.

To examine the specificity of CPuORF responses to abiotic stress, we performed a set of additional experiments. Firstly, we checked to see if water limitation or heat stress had a direct impact on LUC activity by testing stress responses in *35S:LUC* control plants. In neither case was LUC activity significantly different from controls (Figure 4). Next, we examined the effect of mannitol and heat on the function of the well‐characterised *SUPPRESSOR OF ACAULIS 51* (*SAC51*; *At5g64340*) CPuORFs that respond to the polyamine thermospermine (Imai et al., 2006). As expected, *35S:SAC51_CPuORF‐LUC* plants showed an increase in LUC activity after thermospermine treatment (while the same treatment was inhibitory to *35S:LUC* and *35S:CPuORF47‐LUC* activity; Figure 4c) but showed no significant response to abiotic stress treatments (Figure 4a,b). Together, the data suggest that the response of CPuORF19 and CPuORF47 to drought stress and CPuORF46 to increased temperature are not general stress responses and may be specific.

### 3.5 The mORF of the CPuORF47 transcript protects against drought

**Figure 5:**
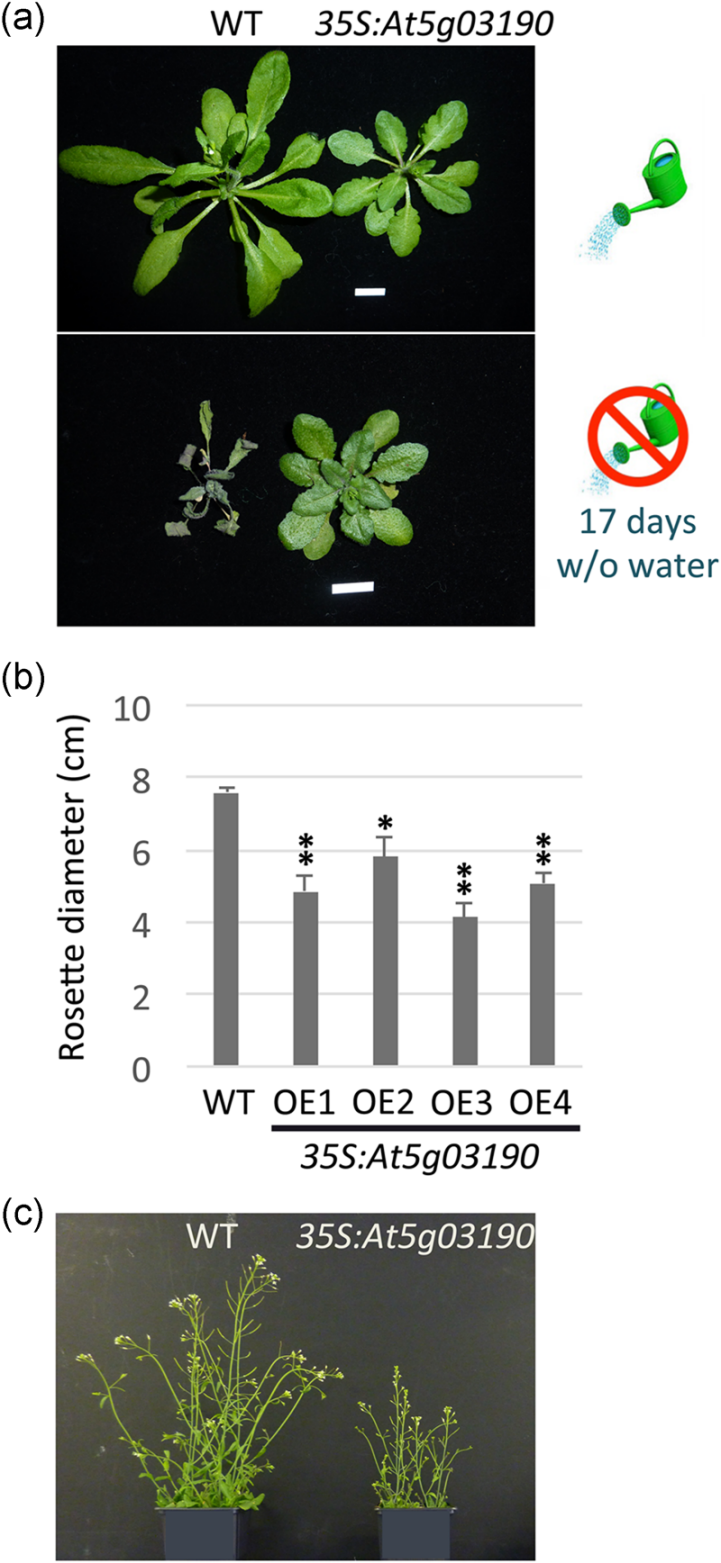
Constitutive expression of the CPuORF47 mORF enhances drought tolerance. (a) Comparison of whole plant phenotypes following drought treatment (bottom) relative to well‐watered controls (top) for WT and 35S:*At5g03190*. Plants are approximately 4 weeks old. (b) Mean rosette diameter for 4‐week‐old WT and 35S:*At5g03190* (four independent lines OE1–4) plants under control conditions. Data represents mean ± SEM (*n* = 8). Significant differences to WT are indicated (*p < 0.05 and **p < 0.01; Tukey HSD inference). (c) 35S:*At5g03190* plants (right) show a reduced stature compared to WT (left) under standard growth conditions. Images in (a) and (c) are representative of the four independent lines, OE1–4. CPuORF, conserved peptide upstream open reading frame; HSD, honestly significant difference; mORF, major open reading frame; WT, wild‐type [Color figure can be viewed at wileyonlinelibrary.com]

Above, we show that CPuORF47 is a regulatory element that responds to water limitation, suggesting that translation of its mORF (*At5g03190*), which encodes a predicted methyltransferase of unknown function, may confer a measure of drought tolerance. To examine this, we constitutively expressed *At5g03190*, without its 5′‐leader, from the 35S promoter, and examined whether the plants showed enhanced drought resistance. After 17 days without watering, 4‐week‐old *35S:At5g03190* plants remained green and healthy, whereas wild‐type plants under the same conditions demonstrated significant wilting (Figure 5a). However, under well‐watered conditions *35S:At5g03190* plants showed growth retardation (which may partially explain their drought tolerance; Figures 5 and S6) and reduced reproductive capacity (Figure S6) compared to wild‐type controls, indicating that uncontrolled expression of *At5g03190* is detrimental to plant health.

## 4 DISCUSSION

### 4.1 CPuORFs respond rapidly to change, maintaining homeostasis

Several plant CPuORFs have been found to be responsive to different signals (reviewed van der Horst et al., 2020). Importantly, the applicability of conditional CPuORFs for crop improvement has been the focus of recent research (Xing et al., 2020; Xu et al., 2017; Zhang et al., 2018). Adding to that body of work, we show that CPuORF19 and CPuORF47 both respond to water limitation, while CPuORF46 responds to increased temperature (Figure 4), suggesting that HG7a and HG17 CPuORFs may be useful tools in the development of stress‐tolerant crops. Interestingly, these CPuORFs have been conserved in lineages that pre‐date angiosperms and are at least 350 million years old (Lloyd & Davies, 2013; Sessa et al., 2014; Takahashi et al., 2020), suggesting that these ancient regulatory elements have possibly contributed to stress responses throughout land plant evolution.

Mutations in animal uORFs, including conserved uORFs, results in the deregulation of associated mORF translation, which in some cases is linked to disease (Jürgens et al., 2021; Wethmar et al., 2010). Thus, the role of these uORFs is to maintain homeostasis by preventing inappropriate mORF translation that might result in harmful phenotypes. Interestingly, a small number of plant CPuORFs has been shown to prevent the uncontrolled translation of adjacent mORFs that may lead to fitness costs under normal conditions (Hanfrey et al., 2002; Ribone et al., 2017; Thalor et al., 2012; Xu et al., 2017). Similarly, we speculate that the CPuORF of *At5g03190* (CPuORF47) acts to suppress the expression of its mORF, which is detrimental to plant health under normal conditions (Figures 5 and S6). Misexpression of regulatory genes in plants often incurs costs to growth and fecundity and is a significant challenge in the development of crops resistant to biotic or abiotic stress (Da Silva et al., 2019), hence the recent interest in the application of CPuORFs in crop protection. CPuORFs, which may have evolved to mitigate against uncontrolled mORF expression to maintain homeostasis, act at the level of translation independently of transcription, providing an additional layer of regulation that allows rapid responses to a dynamically changing environment.

### 4.2 Conserved CPuORF C‐terminus correlates with stalling during translation termination

Understanding the structural organisation and mode of action of these conditional CPuORFs will be important for realising the potential of these regulatory elements as tools with utility both in the laboratory and in the field. Across taxonomic domains, the prevalence of C‐terminal conservation amongst CPuORF peptides is striking and suggests that these peptides regulate the efficiency of translation termination (Dever et al., 2020). We have shown that the conserved C‐terminus of CPuORF47 is required for its inhibitory function and that this region of HG17 CPuORFs includes ribosome arrest sequences (Figures 2 and 3), while its divergent N‐terminus may play a role in conditional responses (Figure 4 and Figure S5). Interestingly, we found that half of the CPuORFs with a conserved C‐terminus (class I and class IIb) potentially cause stalling during translation termination. For the remaining CPuORFs studied here, the evidence for ribosome stalling is not clear, suggesting that they either attenuate translation passively or stall ribosomes outside of the C‐terminus, during translation elongation. To date, there are few examples of eukaryotic uORFs that stall in the elongation, rather than termination, phase of translation, suggesting that this mode of action is less common (see Hayashi et al., 2017). Given our findings and the limited positional information currently available for CPuORF‐mediated eukaryotic ribosome stalling, it will be interesting to look at the relative frequencies of the two mechanisms.

### 4.3 Future prospects

There is a growing list of CPuORFs that either enhance or suppress translation of an associated mORF, in a range of specific conditions such as intracellular metabolite levels and abiotic and biotic stresses (summarised in Table S1). However, it is only recently that the potential of conditional CPuORFs for crop improvement has been explored (Xing et al., 2020; Xu et al., 2017; Zhang et al., 2018), although only a handful of CPuORFs reportedly respond to agriculturally relevant conditions (Bazin et al., 2017; Xu et al., 2017; Zhu et al., 2012; Table S1). Here, we identify uncharacterised Arabidopsis CPuORFs that respond to abiotic stresses linked to water limitation (Figure 4). We have only investigated a limited number of conditions, but expect that, as more conditions are tested, new conditional CPuORFs will be identified. Although a significant number of plant CPuORFs have been identified (see Table S1), finding these elements is not straightforward. In silico searches for plant CPuORFs have tended to focus on those containing a canonical AUG initiation codon (van der Horst et al., 2019). However, a small number of non‐AUG plant CPuORFs have been identified (Laing et al., 2015; van der Horst et al., 2019), suggesting that more CPuORFs remain to be discovered. Identification of *bona fide* CPuORFs with high confidence will require a combination of approaches, including in silico searches (e.g. Takahashi et al., 2020; van der Horst et al., 2019), proteomics and functional information, including ribo‐seq data, which have proved successful in other systems (reviewed Chen & Tarn, 2019). In addition, appropriate use of conservation criteria, such as consideration of evolutionary distances over which uORFs are conserved, will be essential for identification of functional CPuORFs (e.g., see Table S1).

As all the information required for ribosome stalling and conditional responses is contained within the short CPuORF coding region, these elements are ripe for molecular engineering and synthetic biology approaches. Already, CRISPR/Cas9 genome editing has been employed to modify plant uORF sequences in situ, thereby manipulating translation (Si et al., 2020; Um et al., 2021; Zhang et al., 2018).

The potential to generate novel regulatory uORFs, capable of sensing specific signals and controlling the translation of any gene of choice accordingly, indicates the broad applicability of these versatile elements.

## Supporting information

Supporting information.

Supporting information.

Supporting information.

Supporting information.

Supporting information.

Supporting information.

Supporting information.

Supporting information.

Supporting information.
