# Supplementary material for: Plants utilise ancient conserved peptide upstream open reading frames in stress‐responsive translational regulation
Source: Plant Cell Environ. 2022 Feb 15;45(4):1229–41. doi: 10.1111/pce.14277 (PMC9305500; doi:10.1111/pce.14277)
Supplement: Supplementary file 1 — Supporting information. [file PCE-45-1229-s001.pdf]

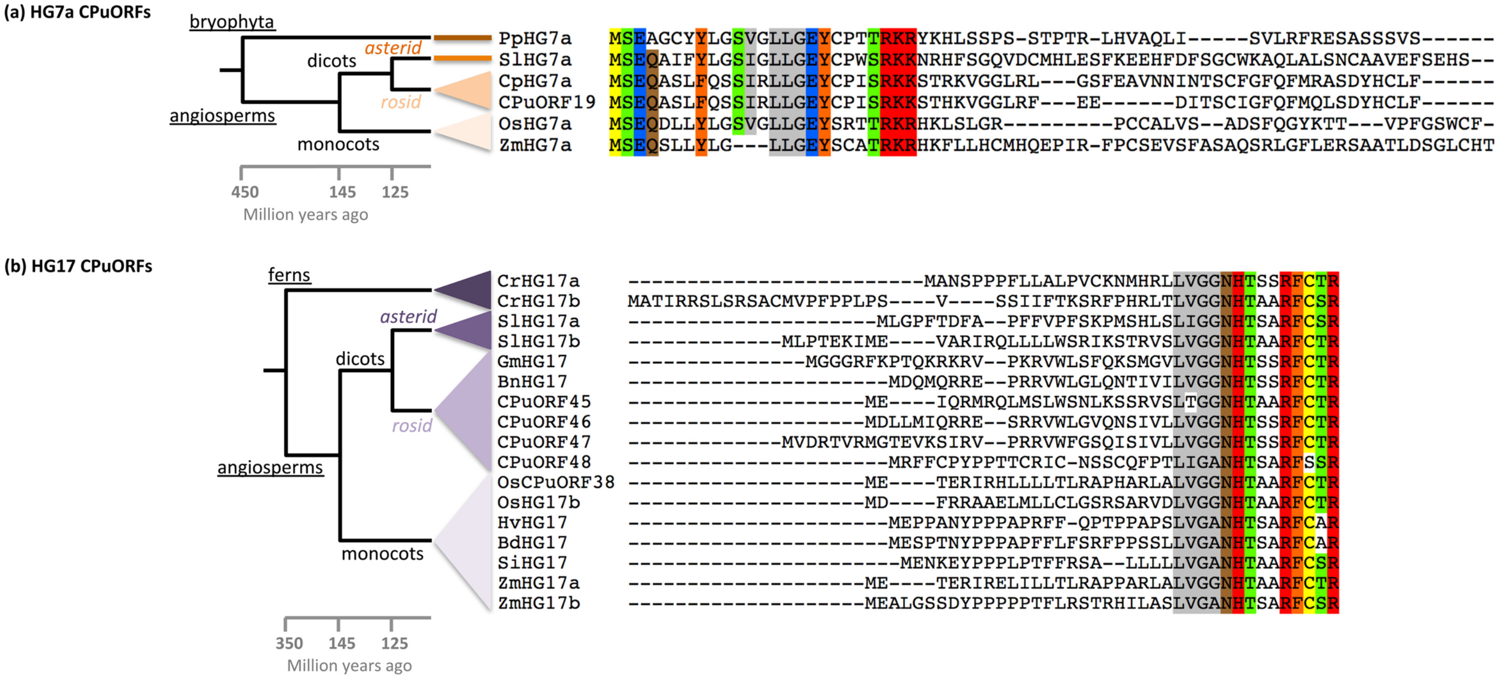

Figure S1 Plant CPuORFs are ancient in origin. Clustal Omega alignment of (a) HG7a and (b) HG17 CPuORF peptide sequences. In both (a) and (b) phylogenetic trees show the range of lineages in which the CPuORFs have been identified, and the estimated divergence times (million years ago). Species abbreviations: *Arabidopsis thaliana* (CPuORF45-48), *Brachypodium distachyon* (Bd), *Brassica napus* (Bn), *Carica papaya* (Cp), *Ceratopteris richardii* (Cr), *Glycine max* (Gm), *Hordeum vulgare* (Hv), *Oryza sativa* (OsCPuORF38 and OsHG17b), *Physcomitrium patens* (Pp), *Setaria italica* (Si), *Solanum lycopersicum* (Sl) and *Zea mays* (Zm).
