# Supplementary material for: Plants utilise ancient conserved peptide upstream open reading frames in stress‐responsive translational regulation
Source: Plant Cell Environ. 2022 Feb 15;45(4):1229–41. doi: 10.1111/pce.14277 (PMC9305500; doi:10.1111/pce.14277)
Supplement: Supplementary file 2 — Supporting information. [file PCE-45-1229-s002.pdf]

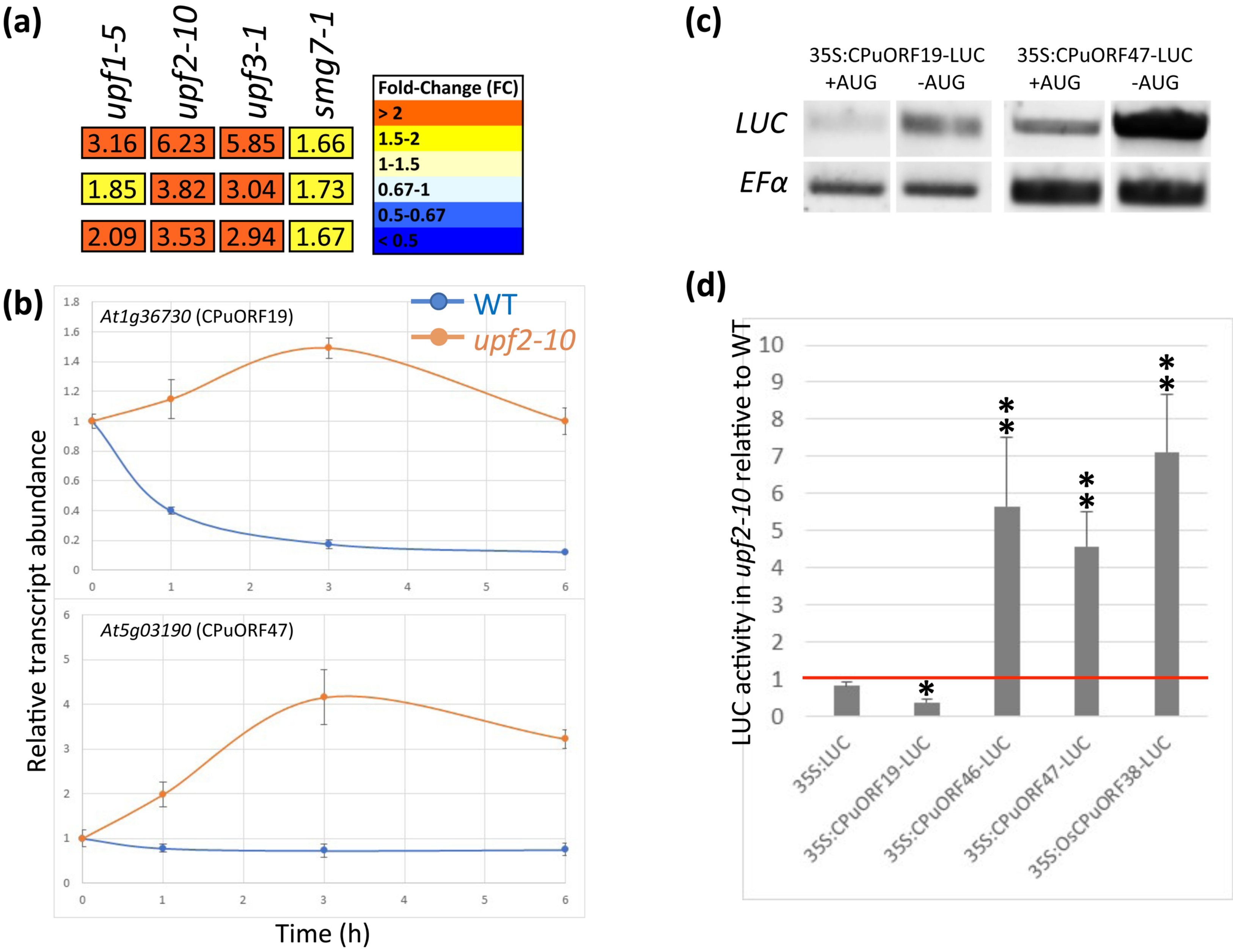

Figure S2 CPuORFs partially function through the NMD pathway. (a) Microarray analysis of transcript abundance for CPuORF19, CPuORF46 and CPuORF47-containing transcripts in various NMD mutants. Numbers represent fold-change values in mutants relative to WT. Data for *upf1-5*, *upf3-1* and *smg7-1* mutants is from Rayson et al. (2012) [https://doi.org/10.1371/journal.pone.0031917]. Data for *upf2-10* was previously unpublished. (b) Stability of CPuORF19 and CPuORF47-containing transcripts over time in WT and *upf2-10* NMD deficient plants. Leaves were harvested from plants and vacuum infiltrated with 300µg/ml (w/v) cordycepin transcription inhibitor. Time 0h samples were immediately flash frozen. The remaining samples were incubated at room temperature for the appropriate time (1h, 3h and 6h), after which samples were flash frozen. Total RNA was extracted from all samples, and RT-qPCRs performed. Abundance was normalised to the *ACTIN2* transcript. The two plots show that, over time, transcript abundance in WT samples (blue line) declines, consistent with transcript decay. In contrast, transcript abundance appears to be stable in *upf2-10* samples (orange line), suggesting that decay in WT is due to NMD. (c) CPuORF-LUC transcript stability relies on translation of the CPuORF sequence. Total RNA was isolated from leaves of transgenic Arabidopsis plants transformed with mutant CPuORF (-uAUG) reporters, relative to the appropriate WT control (CPuORF +uAUG), for both 35S:CPuORF19-LUC and 35S:CPuORF47-LUC lines. Semi-quantitative RT-PCR reactions were performed to amplify the LUC gene and compared to the housekeeping gene *αEF1*. Increased transcript abundance in -uAUG samples, where the CPuORF is not translated, relative to the +uAUG samples, suggests that stability of the *LUC* transcript is affected by CPuORF translation. The CPuORF19 and CPuORF47 PCRs were run on separate gels, but in each case bands are from the same gel (image colour inverted to aide viewing of weaker bands) with intervening lanes removed for clarity. (d) LUC activity measured in leaves of transgenic *upf2-10* NMD-deficient Arabidopsis plants transformed with various 35S:CPuORF-LUC constructs, compared to WT plants carrying the same constructs. Means ± SEM are shown. For *upf2-10* plants with CPuORF-LUC the number of independent lines (*n*)=20, CPuORF46-LUC *n*=20, CPuORF47-LUC *n*=16, and for OsCPuORF38-LUC *n*=16. As control LUC activity was measured in WT and *upf2-10* plants transformed with a 35S:LUC construct (WT *n*=63; *ufp2-10 n*=21). Significant differences between WT and *upf2-10* lines for each CPuORF at p<0.05 and p<0.01 (Tukey HSD inference) are represented by a single or double asterisk, respectively. The data indicates that HG17 CPuORFs might function at least in part via NMD, and that this is evolutionarily conserved from monocots to dicots. Surprisingly, although the CPuORF19 transcript is an NMD target, LUC activity in *upf2-10* mutants was reduced relative to wild-type controls, suggesting that the CPuORF19 may not be the key determinant that triggers its transcript to NMD. Analysis of the predicted CPuORF mRNA reveals a long 3'-UTR (~500bp), which is also known to induce NMD in plants.
