# Supplementary material for: Plants utilise ancient conserved peptide upstream open reading frames in stress‐responsive translational regulation
Source: Plant Cell Environ. 2022 Feb 15;45(4):1229–41. doi: 10.1111/pce.14277 (PMC9305500; doi:10.1111/pce.14277)
Supplement: Supplementary file 3 — Supporting information. [file PCE-45-1229-s008.pdf]

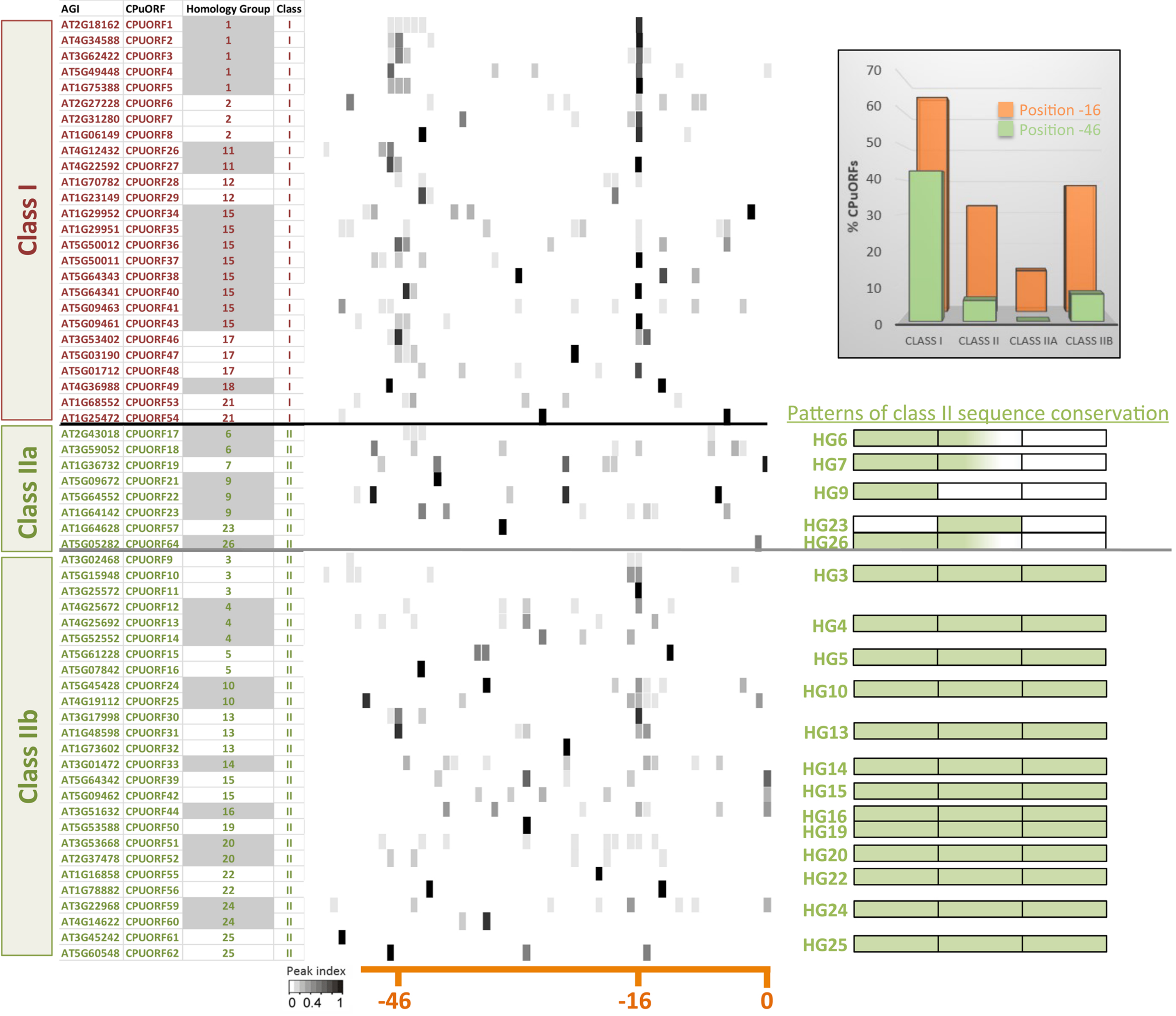

Figure S3 The distribution of PARE reads in Arabidopsis class I and class II CPuORFs. Clustered heat map of PARE reads redrawn from previously published analyses (Hou et al., 2016 - doi.org/10.1105/tpc.16.00295). The first nucleotide of the CPuORF stop codon is position 0. Black/grey blocks represent the peak index value (calculated by dividing the number of PARE reads at a particular position by the number of total reads in a 31-nucleotide flanking region; Hou et al., 2016), with darker colours representing a greater accumulation of reads. AGI, CPuORF number, homology group and CPuORF class, is listed to the left. To highlight the distinct patterns of PARE read accumulation seen for class I and class II CPuORFs, class I are at the top of the heat map (in red), with class II at the bottom (in green). These class II CPuORFs can be further subdivided into class IIa and class IIb, based on different patterns of conservation as shown to the right (green fill indicates peptide sequence conservation). The bar chart (inset), shows the proportion of class I, all class II, class IIa and classIIb CPuORFs that accumulate PARE reads at positions -16 (orange bars) and -46 (green bars), relative to the CPuORF stop codon (position 0). Threshold peak index >0.1.
