# Supplementary material for: Plants utilise ancient conserved peptide upstream open reading frames in stress‐responsive translational regulation
Source: Plant Cell Environ. 2022 Feb 15;45(4):1229–41. doi: 10.1111/pce.14277 (PMC9305500; doi:10.1111/pce.14277)
Supplement: Supplementary file 4 — Supporting information. [file PCE-45-1229-s007.pdf]

(a)

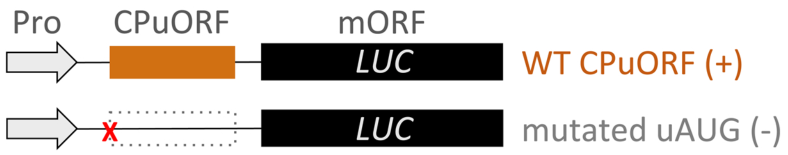

(b)

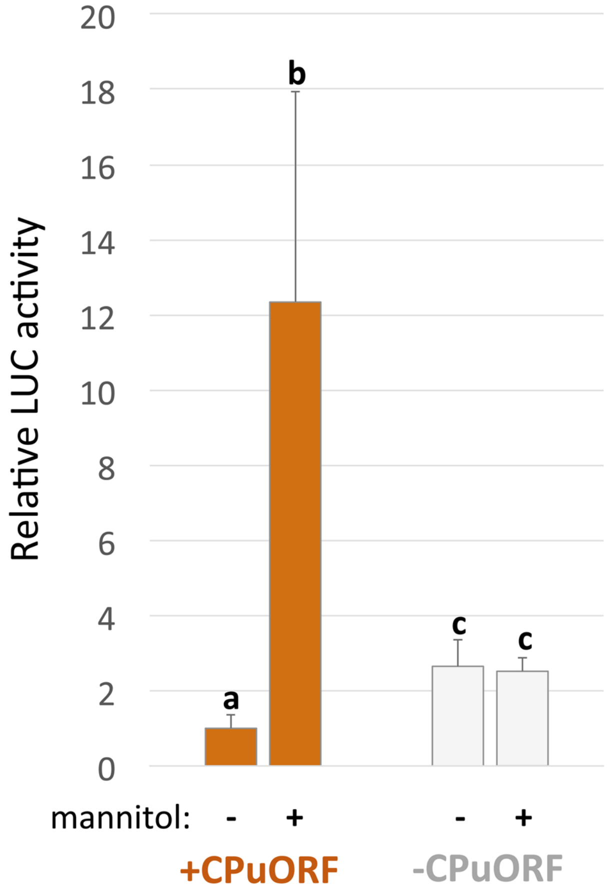

	mock	mannitol
35S:CPuORF47-LUC	(A) 17.35±6.24	(B) 214.21±97.33
35S:(-AUG)CPuORF47-LUC	(C) 45.99±12.07	(D) 43.69±6.21
no. independent lines = 6		
Tukey HSD inference	AvB	** p<0.01
	AvC	ns
	AvD	ns
	BvC	* p<0.05
	BvD	* p<0.05
	CvD	ns

Figure S4 (a) Summary of constructs used. Arrows represent the *35S* promoter. Lines represent the 5'-UTR. The black box represents the major open reading frame (mORF), which encodes the luciferase (LUC) reporter. The coloured box represents CPuORF47. The dotted box represents CPuORF47 where the start codon (uAUG) has been mutated (red cross) to prevent its translation, releasing the inhibition of mORF translation. (b) LUC activity measured in leaves from Arabidopsis transformed with the CPuORF47-containing reporter constructs. Activity of CPuORFs with a mutated uAUG (-CPuORF; grey bars) is presented relative to WT CPuORFs (+CPuORF; orange bars), following mock (-) or mannitol (+) treatment of leaf samples. Mean fold-changes ± SEM are shown. Bars with different letters are significantly different from one another (Tukey HSD inference; $p<0.05$). Note that translation of CPuORF47 attenuates that of the mORF (LUC) and is also required for the response of CPuORF47 to mannitol. Data used to generate the chart is presented in the table below.
