# Supplementary material for: Plants utilise ancient conserved peptide upstream open reading frames in stress‐responsive translational regulation
Source: Plant Cell Environ. 2022 Feb 15;45(4):1229–41. doi: 10.1111/pce.14277 (PMC9305500; doi:10.1111/pce.14277)
Supplement: Supplementary file 5 — Supporting information. [file PCE-45-1229-s005.pdf]

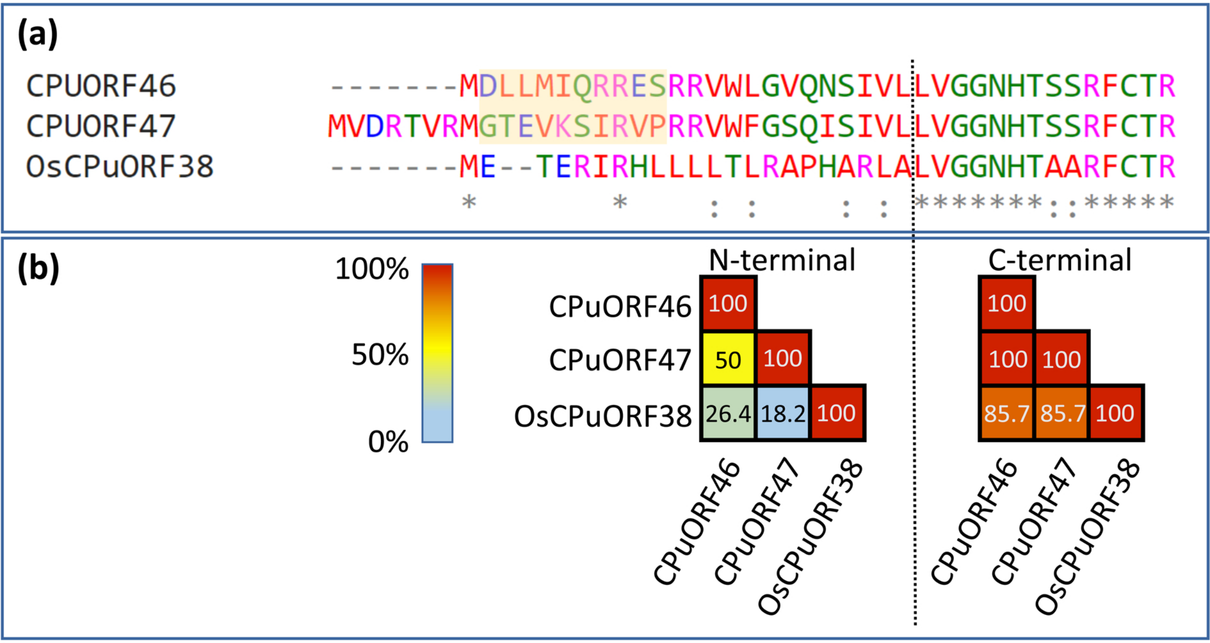

Figure S5. (a) Clustal Omega alignment of Arabidopsis CPuORF46, CPuORF47 and rice OsCPuORF38 peptide sequences. The yellow box highlights a block of residues that differ between CPuORF46 and CPuORF47. Also note the N-terminal extension of CPuORF47. These may explain the difference in conditional responses seen for these two CPuORFs, which needs further testing. (b) Heat maps of percentage amino acid identities shared between the N-terminal (left) and C-terminal (right) regions of HG17 CPuORFs from Arabidopsis and rice. % Identities are also shown.

The dotted line in (a) through (b) separates the conserved C-terminal and the divergent N-terminal sequences.
