# Supplementary material for: Plants utilise ancient conserved peptide upstream open reading frames in stress‐responsive translational regulation
Source: Plant Cell Environ. 2022 Feb 15;45(4):1229–41. doi: 10.1111/pce.14277 (PMC9305500; doi:10.1111/pce.14277)
Supplement: Supplementary file 6 — Supporting information. [file PCE-45-1229-s003.pdf]

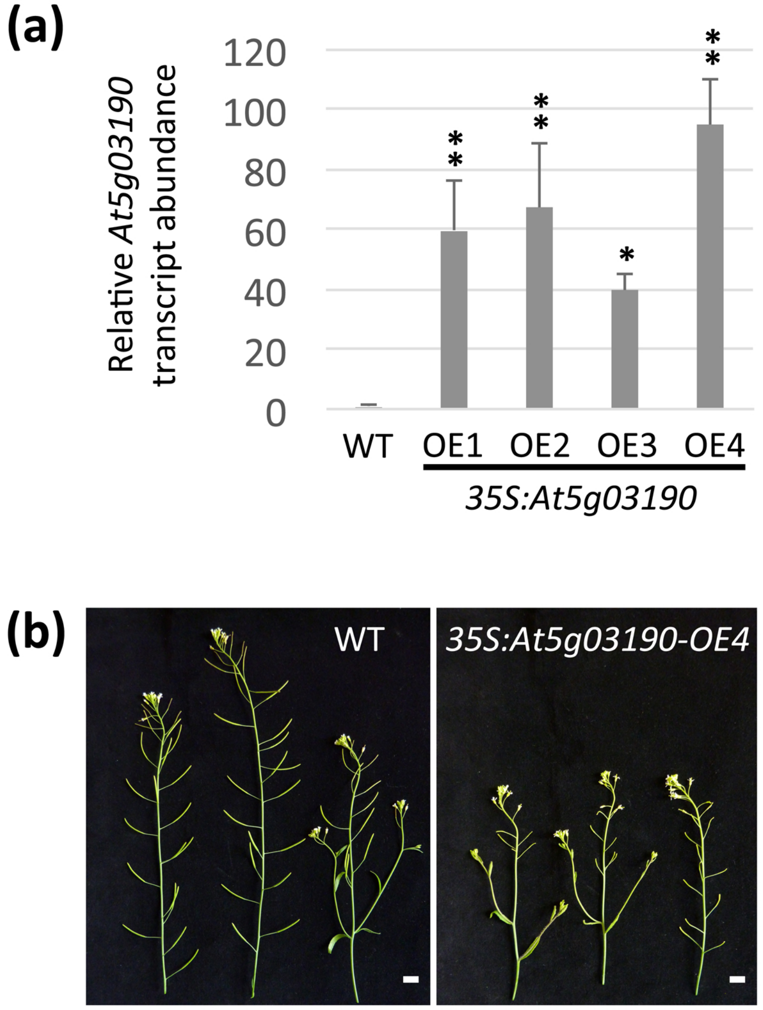

Figure S6 Constitutive expression of the *At5g03190* mORF results in an adverse phenotype. (a) Quantitative RT-PCR analysis of *At5g03190* expression in over-expressor lines. Transcript abundance normalised to *ACTIN2* transcript is shown for four over-expressor lines (OE1-4) relative to non-transgenic wild-type (WT) plants (mean of 3 independent samples with 3 technical replicates ± standard error of the mean). Significant differences to WT are indicated ($p<0.05$ (*) and $p<0.01$ (**); Tukey HSD inference). (e) 35S:*At5g03190* OE4 plants (right) produce aborted siliques, compared to WT plants (left), under well-watered (control) conditions.
